# Abscess drain migration into the colon following laparoscopic cholecystectomy

**DOI:** 10.1016/j.jimed.2023.07.001

**Published:** 2023-07-07

**Authors:** Daniel K. Derrick, Noah Fanous, Anne Wells, Jorge Lopera

**Affiliations:** Department of Radiology, UT Health San Antonio, San Antonio, TX, USA

## Abstract

Percutaneous abscess drainage is a procedure commonly performed by interventional radiologists to provide source control on infections using CT or ultrasound guidance. The interventionalist has many different sizes and shapes of catheters to treat abscesses of varying sizes and locations, but the general approach to each abscess is similar: provide a percutaneous route for purulence, bacteria, necrotic tissue, and other debris to escape the body. While generally considered a low-risk procedure, adverse events can occur due to operator error or other means. We present a unique case of an abscess drain placed into a right upper quadrant abscess that formed following laparoscopic cholecystectomy that perforated and entered the colon. Astute physicians, both in the emergency department and the radiology reading room, were able to rapidly rule out more common post-operative complications and make the correct diagnosis, likely preventing dangerous sequelae from developing in this patient.

## 1 Introduction

Laparoscopic cholecystectomy continues to be the standard of care for gallbladder disease, and the etiology behind adverse sequelae is yet unknown, given the spectrum of potential complications that result from this procedure. Complications occur in less than 5% of cases and most commonly include additional common bile duct stones, bile leak, superficial wound infection, abscess formation, and bile duct injury.^1^^,^^2^

In the event of an abscess, percutaneous abscess drainage is routinely used for simple, unilocular abscesses, and reduces rates of mortality drastically.^3^ Percutaneous abscess drains have a high rate of success, around 80%.^4^ These procedures are the first line for intra-abdominal abscesses^5^ and are often used to avoid additional surgical intervention and prevent morbidity and mortality due to sepsis. Using imaging guidance, typically CT or ultrasound, an experienced interventionalist can access and aspirate the abscess in a short amount of time, typically under 30 minutes. The physician can then choose to leave a percutaneous drain in the abscess to promote continued draining and prevent reformation of the abscess.

## 2 Case presentation

A 54-year-old male with a history of GERD, HTN, and gastritis is admitted following three days of worsening vomiting, nausea, reflux, and mid-epigastric pain. He also reports multiple episodes of “greenish” tinged vomitus and has been unable to keep solid food down. He has subsequently developed severe burning reflux and mid-epigastric pain. Acute cholecystitis was diagnosed, laparoscopic cholecystectomy was performed, and the patient reported one-week later to the ED with subjective complaints of chills and night sweats in addition to mid-epigastric pain.

On physical exam, the patient is awake, alert and in no acute distress. His abdomen is soft, non-distended, non-tender to palpation and has decreased bowel sounds present. The surgical wound area is not tender to touch. There are no other laceration abrasions, or ecchymosis present.

**Fig. 1:**
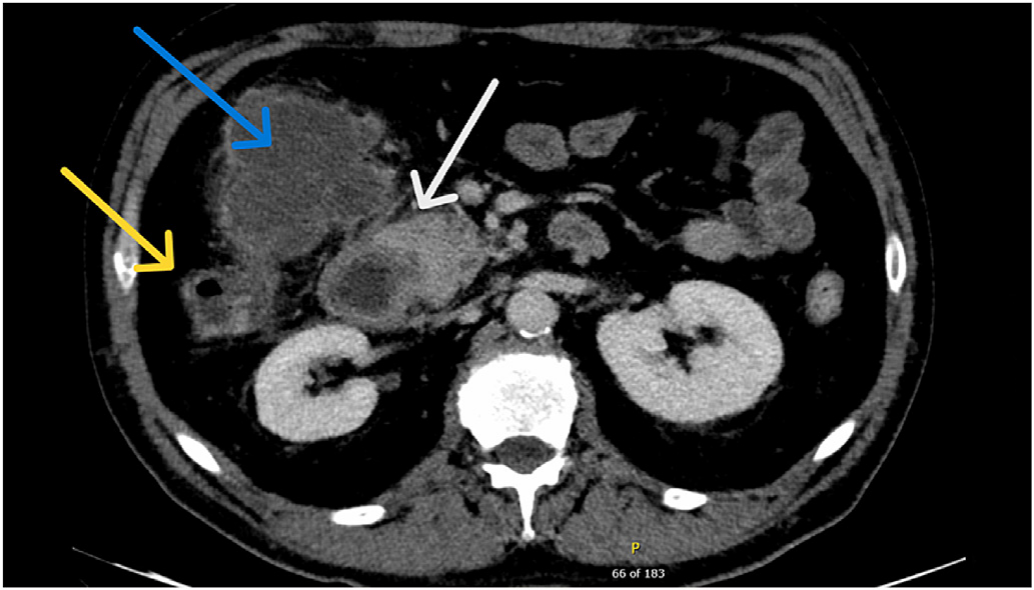
Axial CT angiogram of the abdomen in the arterial phase demonstrating large rim-enhancing fluid collection (blue arrow) in the right upper quadrant consistent with abscess. This collection abuts both the colon (yellow arrow) and duodenum (white arrow). There is no visualized site of bowel perforation, although this is not excluded.

**Fig. 2:**
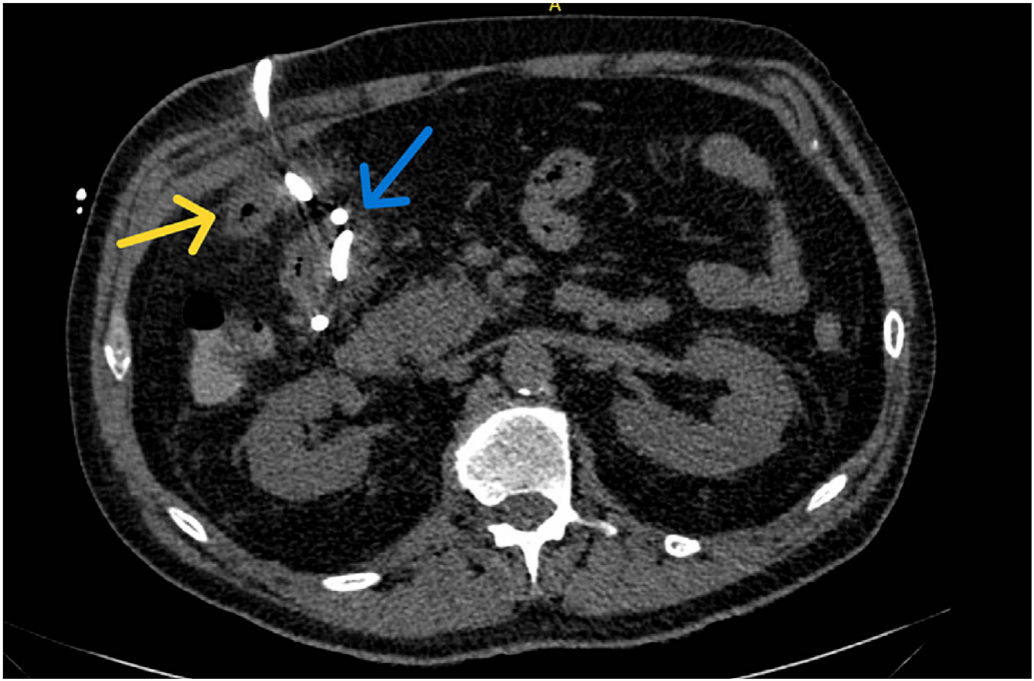
Axial non-contrast CT abdomen after treatment showing post-surgical changes of cholecystectomy and right upper quadrant abscess pigtail drain placement (blue arrow) with interval decreased size of the previously seen right upper quadrant abscess which closely abuts the hepatic flexure of the colon (yellow arrow).

Pertinent lab results include lipase: >30,000 (elevated), total bilirubin: 2.1 (elevated), leukocytes: 13.7 (elevated). The patient was admitted to the hospital, and a CT scan showed a 4.8 cm by 4.0 cm intraabdominal abscess (see Fig. 1 below) into which a percutaneous drain was placed under ultrasound guidance (see Fig. 2).

**Fig. 3:**
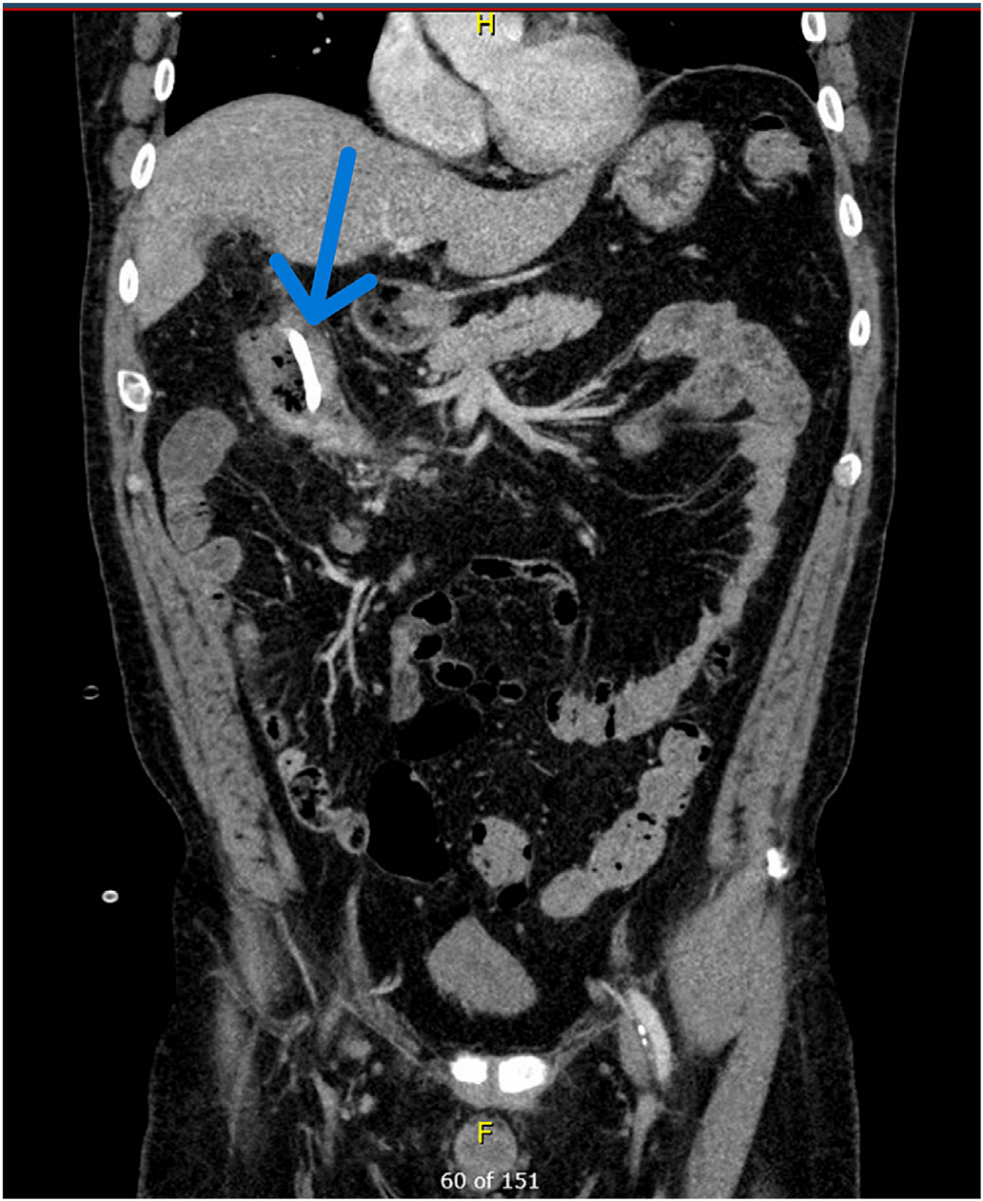
Coronal non-contrast CT abdomen showing abscess catheter within the colonic lumen (blue arrow) approximately 2 weeks after placement. Previously seen abscess is otherwise not well seen on this exam.

Two weeks after drain placement, the patient is seen in clinic for follow up. He is tolerating a regular diet and is flushing his drain daily with sterile water. The patient's vital signs are within normal limits. On physical exam there is no erythema or induration at the site of the drain coming out of skin at the right costal margin. There is a small amount of purulent material coming from the drain. Bowel sounds are present, and the abdomen is not distended. The patient is scheduled for a follow up CT scan to assess the size of the abscess before another clinic appointment to potentially remove the drain in one week. The CT is shown in Fig. 3.

## 3 Discussion

This patient's presentation was complex yet typical due to the often-complex sequelae of adverse events following laparoscopic cholecystectomy. A high degree of clinical suspicion for acute pancreatitis was warranted in this case, but complications following invasive procedures as described here are more probable and may overlap considerably in presentation.

The differential for this presentation includes:

1. Colon perforation (CP). Gallstones left in the peritoneal cavity post-laparoscopic cholecystectomy cause intra-abdominal abscesses in 1–12% of patients,^1^ with pigmented gallstones being more likely to cause complications. Gallstone spillage commonly remains asymptomatic, with only 0.04–19% of cases causing clinical symptoms.^2^ Colonic injury and/or perforation following percutaneous drainage is an exceptionally rare (<1% incidence) complication of nephrolithotomy and cholecystectomy.
2. Sepsis secondary to abscess. Patients with abscesses can present with tachycardia, prolonged ileus, dehydration, oliguria, tachypnea, respiratory alkalosis, fever, anorexia, and abdominal pain. However, there may be only minimal symptoms, such as mild liver dysfunction, mild fever, or prolonged ileus in patients with retroperitoneal or deep pelvic abscesses. Untreated abdominal abscesses can furthermore lead to sepsis. Sepsis presents with fever, tachycardia, and hypotension and can lead to distributive shock and acute respiratory distress syndrome.
3. Acute Pancreatitis (AP). Patients with AP typically present with severe upper abdominal pain located in the epigastric region and left upper quadrant. Digestive enzymes are unable to be secreted and begin to leak around the pancreas and into systemic circulation. This results in a serum amylase and lipase rise within 12 hours of pancreatitis onset. Physical exam findings range from mild epigastric tenderness with few systemic symptoms to diffuse, severe abdominal pain, distention, scleral icterus, fever, periumbilical ecchymosis, hypoxemia, hypotension, and hypoactive bowel sounds. Imaging would show a diffusely enlarged pancreas, potentially with peripancreatic fluid, fat suppression, and blurred margins.

Diagnosis: The diagnosis for this case is an abscess drain migrated into the colon. Percutaneous drainage is the first-line therapy for the treatment of intra-abdominal abscesses^1^ with a success rate of 70% as a single treatment.^2^ Complications occur in less than 5% of cases and most commonly include additional common bile duct stones, bile leak, superficial wound infection, and bile duct injury. Abscess drain migration is a rare complication usually associated with gallbladder perforation and spillage of stones into the peritoneal cavity.^4^

## 4 Conclusions

Imaging at four weeks shows the tip of the drain in the right colon with shrinkage and eventual resolution of the abscess. The decision was made to leave the drain in place, allowing for the created tract to mature, and it was removed 6 weeks after placement without incident. The patient had no fistula formation or additional sequelae due to the abscess drain.

In this unique case of colonic perforation due to drain migration, allowing the drain to remain in place prevents any additional sequelae that can often result from bowel trauma.

## Ethical approval

The study was approved by the ethics committee of the UT Health San Antonio. All clinical practices and observations were conducted in accordance with the Declaration of Helsinki.

## Patient consent

Written informed consent was obtained from the patient for publication of the case report and any accompanying images.

## Declaration of competing interest

All authors have no conflicts of interest to declare.
